# Long-term use of biologic agents does not increase the risk of serious infections in elderly patients with rheumatoid arthritis

**DOI:** 10.1007/s00296-016-3631-z

**Published:** 2016-12-20

**Authors:** Hirotoshi Kawashima, Shin-ichiro Kagami, Daisuke Kashiwakuma, Kentaro Takahashi, Masaya Yokota, Shunsuke Furuta, Itsuo Iwamoto

**Affiliations:** Research Center for Allergy and Clinical Immunology, Asahi General Hospital, I-1326, Asahi City, Chiba 289-2511 Japan

**Keywords:** Rheumatoid arthritis, Biologic agents, Infection, Aging, Glucocorticoid

## Abstract

This study aimed to determine whether the long-term use of biologic agents increases serious infections in elderly patients with rheumatoid arthritis (RA) and to determine the risk factors of serious infections in biologics-treated elderly RA patients. We retrospectively analyzed the incidence rate of serious infections that required hospitalization between biologics-treated and non-biologic disease-modifying antirheumatic drug (DMARD)-treated elderly RA patients (aged over 65 years). We examined the risk factors for serious infections in biologics-treated elderly RA patients. We found that, during a 3-year observation period, the incidence rate of serious infections was not significantly different between biologics-treated and non-biologic DMARD-treated elderly RA patients [8.0 (95% CI 4.7–13.5) and 6.3 (95% CI 4.1–9.5) events per 100 person-years of follow-up, respectively, *P* = 0.78]. The time to the first serious infection did not significantly differ between the two groups by the analysis of the Kaplan–Meier curves, either (*P* = 0.46). We then found that prednisolone doses alone were significantly associated with serious infections in biologics-treated elderly RA patients. Furthermore, we found that prednisolone at 1–4 mg/day was associated with serious infections in biologics-treated patients, but not non-biologic DMARD-treated patients. On the other hand, prednisolone at greater than 5 mg/day was associated with serious infections in both biologics-treated and non-biologics-treated patients. We show that there is not a significant difference between the incidence of serious infections between biologics group and non-biologics group in elderly RA patients (≧65 years) and that even very low-dose glucocorticoid use (prednisolone 1–4 mg/day) is a risk factor for serious infections in biologics-treated elderly RA patients.

## Introduction

Methotrexate (MTX) is an immunosuppressive non-biologic disease-modifying antirheumatic drug (DMARD), which is used as a first-line drug for the treatment of rheumatoid arthritis (RA) [1, 2]. It has also been shown that long-term use of MTX does not appear to be a risk factor for serious infections in RA patients [3–6]. Biologic agents that block the effects of pro-inflammatory cytokines such as tumor necrosis factor (TNF) and IL-6 are used for the treatment of RA when disease activity can not be controlled with conventional DMARDs including MTX and substantially improve outcomes of RA [1, 2]. However, biologic agents, due to their immunological properties, may increase the risk of serious infections in RA patients. Previous studies have reported that biologic agents increase the risk of serious infections in RA patients [7–10]. On the other hand, other studies have not found an increased risk of serious infections with biologic agents in RA patients [6, 11, 12]. Thus, conflicting information still exists regarding the risk of serious infections with biologic therapy for RA.

Increasing age is also an important risk factor for infections. Aging generally induces age-related immune dysfunction, leading to the increased incidence and severity of infections [13, 14]. Furthermore, previous studies have shown that aging is one of the risk factors for serious infections in RA patients [3, 6] and thus there are concerns that administration of biologic agents to elderly patients with RA may increase serious infections. However, there are only a few studies examining the risk of infections in the treatment of elderly RA patients with biologic agents. Schneeweiss et al. [15] found no significant increase in the incidence rate of serious bacterial infections in elderly RA patients receiving antiTNF therapy as compared with those receiving MTX therapy, although the length of follow-up was a relatively short term (1.3 and 0.6 years, respectively). Galloway et al. [9] found no increased in the elderly compared to younger biologic patients. Therefore, the risk of long-term use of biologic agents for serious infections in elderly RA patients still remains largely unknown.

In this study, in order to determine whether the long-term use of biologic agents increases serious infections in elderly RA patients, we retrospectively analyzed the incidence rate of serious infections that required hospitalization between biologics-treated and non-biologic DMARD-treated elderly RA patients (aged over 65 years). Then, to determine the risk factors of serious infections in biologics-treated elderly RA patients, we examined the background profiles between the patients suffering from serious infections and those without serious infections in biologics-treated elderly RA patients.

## Patients and methods

### Patients and cohort analysis

We retrospectively reviewed electronic medical records of 183 sequential RA patients over the age of 65 years at the start date of follow-up whose therapy with or without biologics started between January 2006 and March 2012 at Asahi General Hospital and analyzed the incidence of serious infections in the biologics group and non-biologics group of the patients. Serious infection was defined as infection requiring admission to the hospital or prolongation of hospitalization. All patients fulfilled the ACR 1987 revised criteria for RA [16]. Our hospital was located in the rural area and had the only rheumatic disease center in the area. Consequently, most of our outpatients were admitted to our hospital when they had serious adverse events including infection. For inclusion, we selected all elderly RA patients over the age of 65 years at the start of treatments. Patients were seen regularly by rheumatologists to assess disease activity and disease severity and received routine clinical management. Observation period of a given patient was for 3 years from the initiation of the biologic or non-biologic therapy or from the initiation of the therapy to the discontinuation of the therapy when it discontinued before the 3-year observation period. Patients who lost follow-up due to moving or voluntary dropout were censored at the point. Patients who died during the observation period were censored at the point. Patients who had the treatment interruption of the biologic or non-biologic therapy were censored at that point. Patients who had the switching from biologic therapy to another biologic therapy were continued to observe in the biologics group. Similarly, patients who had the switching from non-biologic therapy to another non-biologic therapy were continued to observe in the non-biologics group.

Sixty-four elderly RA patients received biologic agents because their disease activities could not be controlled by conventional DMARDs treatment: 36 patients received TNF inhibitors including infliximab, etanercept, adalimumab, golimumab, and certolizumab, 6 patients received tocilizumab, and 22 patients were switched to other biologic agents due to the ineffectiveness or adverse effects of initial biologic agents. One hundred and nineteen elderly RA patients received only DMARDs including methotrexate (MTX) and sulfasalazine. DMARDs were also used in some patients of the biologics group. Glucocorticoids were used in some patients of both the biologics and non-biologics groups. Clinical and laboratory assessment at baseline included erythrocyte sedimentation rate (ESR), C-reactive protein (CRP), rheumatoid factor (RF), and comorbidities (Table 1). This study was approved by the Ethics Committee of Asahi General Hospital and was performed in accordance with the principles of the Declaration of Helsinki.

**Table 1:** Baseline characteristics of elderly RA patients

Characteristic	Biologics (*n* = 64)	Non-Biologics (*n* = 119)	*P*
Age (years, mean ± SD)	73.7 ± 5.1	73.7 ± 5.8	0.92
Female, *n* (%)	50 (78.1%)	83 (69.7%)	0.22
Disease duration (years, mean ± SD)	12.7 ± 9.7	10.9 ± 13.3	0.34
RF positive, *n* (%)	57 (89.1%)	97 (81.5%)	0.18
ESR 60 (mm, mean ± SD)	60.6 ± 33.0	36.7 ± 28.7	<0.001
CRP (mg/L, mean ± SD)	27.9 ± 34.4	13.7 ± 35.6	0.011
Steinbrocker stage (I + II/III + IV)	32/32	71/48	0.23
Comorbidities, *n* (%)			
Coexisting lung disease	21 (32.8%)	31 (26.1%)	0.33
Diabetes mellitus	3 (4.7%)	8 (6.7%)	0.58
Medications, *n* (%)			
Methotrexate	51 (79.7%)	95 (79.8%)	0.98
Other DMARDs	21 (32.8%)	61 (51.3%)	0.016
PSL (mg/day)	1.8 ± 2.5	1.9 ± 3.1	0.78
PSL, any dose (%)	28 (43.7%)	44 (37.0%)	0.37
PSL ≧5 mg/day	12 (18.7%)	27 (22.7%)	0.39
Biologics, *n* (%)			
TNF inhibitors	36 (56.2%)		
Tocilizumab	6 (9.4%)		
Switch of biologics	22 (34.4%)		

*RF* rheumatoid factor, *DAS* disease activity score, *DMARDs* disease-modifying antirheumatic drugs, *PSL* prednisolone

### Statistical analysis

Statistical analysis was performed using JMP software version 9.0 (SAS Institute Japan, Tokyo, Japan). Normally distributed continuous data were analyzed using parametric tests (Student’s *t* test). Non-normally distributed data were analyzed using nonparametric tests (Mann–Whitney *U* test or Spearman’s rank correlation coefficient). Categorical data were analyzed using Chi-square test or Fisher’s exact test. The incidence rates were calculated and compared by the person-years method. Risk factors for serious infections were analyzed by multivariate analysis with the logistic regression model. Data of time to the serious infection were analyzed using the Kaplan–Meier method with log-rank test. *P* values less than 0.05 were considered significant.

## Results

### Baseline characteristics of the biologics and non-biologics group in elderly RA patients

To evaluate the safety of long-term use of biologic agents in elderly RA patients, we retrospectively analyzed the incidence of serious infections that required hospitalization between the biologics (*n* = 64) and non-biologics group (*n* = 119) in elderly RA patients (≧65 years). Baseline characteristics of the two groups are shown in Table 1. There were no significant differences in age (73.7 ± 5.1 vs 73.7 ± 5.8 years), sex (female; 78.1 vs 69.7%) or disease duration (12.7 ± 9.7 vs 10.9 ± 13.3 years) between the biologics and non-biologics groups. There were no significant differences in comorbidities including coexisting lung disease (32.8 vs 26.1%) and diabetes mellitus (4.7 vs 6.7%) between the two groups.

In RA medication, there was no significant difference in methotrexate (MTX) use (79.7 vs 79.8%) between the two groups. On the other hand, other DMARDs including sulfasalazine were used more frequently in the non-biologics group (51.3%) than in the biologics group (32.8%, *P* = 0.016). Prednisolone (PSL) was used similarly in the two groups (1.8 ± 2.5 vs 1.9 ± 3.1 mg/day).

### Incidence of serious infections is not different between the biologics and non-biologics groups in elderly RA patients

We then examined the incidence of serious infections between the biologics and non-biologics groups in elderly RA patients (Table 2). During a 3-year observation period, the numbers of events of serious infections were not significantly different between the biologics and non-biologics groups (13 and 21, respectively). The numbers of patients with ≧1 events of serious infections were not significantly different between the two groups, either. The incidence of serious infections was 8.0 (95% CI 4.7–13.5) and 6.3 (95% CI 4.1–9.5) events per 100 person-years of follow-up in the biologics and non-biologics group, respectively, and was not significantly different between the two groups. The most frequent infection was bacterial pneumonia with 6 infections in the biologics group and 12 infections in the non-biologics group (Table 2). Other common infections were cellulitis with three infections in the biologics group and two infections in the non-biologics group and pyelonephritis with one infection in the biologics group and three infections in the non-biologics group. In this cohort, there was no incidence of mycobacterium tuberculosis infection in either group.

**Table 2:** Incidence of serious infections that required hospitalization in elderly RA patients

Serious infections	Biologics (*n* = 64)	Non-biologics (*n* = 119)	*P*
Number of events	13	21	
Number of patients with ≧1 events	10	16	0.68
Biologics, OR (95% CI)	1.2 (0.5–2.8)	1.0 (ref.)	0.74
Observation period (months), median (IQR)	36 (26–36)	36 (31–36)	0.007
Rate, per 100 Person-years (95% CI)	8.0 (4.7–13.5)	6.3 (4.1–9.5)	0.78
Bacterial pneumonia, *n*	6	12	
Cellulitis, *n*	3	2	
Pyelonephritis, *n*	1	3	
Pneumocystis pneumonia, *n*	1	0	
Gastroenteritis, *n*	1	1	
Bacterial arthritis, *n*	1	1	
Viral infection, *n*	0	2	

*IQR* interquartile range, *ref.* reference

**Fig. 1:**
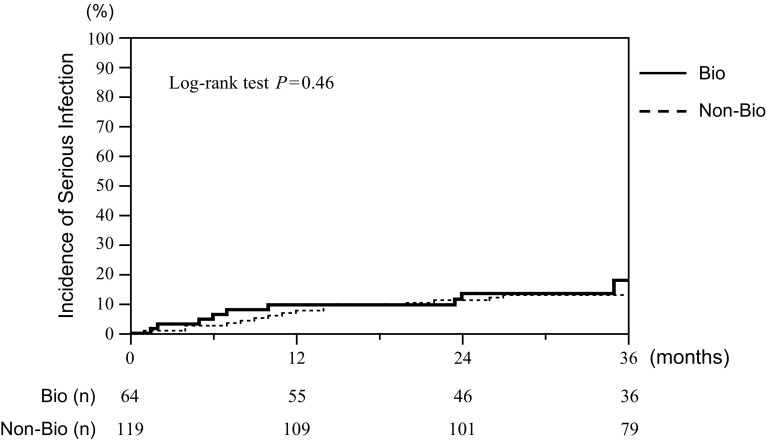
Kaplan–Meier curves of incidence of serious infections between biologics and non-biologics groups in elderly RA patients. Time to the first serious infection that required hospitalization was analyzed using the Kaplan–Meier method. Incidence of serious infections is not significantly different between biologics and non-biologics groups in elderly RA patients (log-rank test *P* = 0.46)

Next, we analyzed the incidence of serious infections between the biologics and non-biologics groups using the Kaplan–Meier method (Fig. 1). The time to the first serious infection did not significantly differ between the two groups (Log-rank test *P* = 0.46). These results suggested that treatment with biologic agents did not significantly increase the incidence of serious infections in elderly RA patients as compared with that with non-biologic agents. In the biologics group, 39 patients, including three patients who restarted biologic agents after recovery from serious infection, continued biologic agents during the observation period.

Of 25 patients who discontinued the treatment with biologic agents, eight patients stopped biologic agents because of remission, seven patients stopped them because of serious infections, six patients stopped them because of insufficient effects, and four patients discontinued them because of the development of malignancies. There were three deaths in the biologics group (one serious infection and one unknown cause), while there were four deaths in the non-biologics group (two serious infection, one lung cancer, and one arrhythmia).

### Risk factors of serious infections in the biologics group of elderly RA patients

To determine the risk factors for serious infections in the biologics group of elderly RA patients, we examined the background profiles between the patients suffering from serious infections and those without serious infections in biologics-treated elderly RA patients (Table 3). There were no significant differences in age (74.5 ± 4.4 vs 73.6 ± 5.3 years) or disease duration (15.4 ± 9.4 vs 12.2 ± 9.7 years) between the patients with and without serious infections. Furthermore, there were no significant differences in the use of MTX (80.0 vs 79.6%) and other DMARDs (20.0 vs 35.2%) between the patients with and without serious infections. In addition, various biologic agents such as TNF inhibitors were used at a similar frequency between the two patients groups (Table 3). However, we found that PSL dose (4.7 ± 3.2 vs 1.3 ± 2.0 mg/day, *P* < 0.001) and PSL use (90.0 vs 35.2%, *P* = 0.001) were significantly higher in the patients suffering from serious infections than those without serious infections (Table 3).

**Table 3:** Risk factors for serious infections in biologics group of elderly RA patients

	Serious infection		*P*
	(+) (*n* = 10)	(−) (*n* = 54)	
Age (years, mean ± SD)	74.5 ± 4.4	73.6 ± 5.3	0.63
Female, *n* (%)	7 (70.0%)	43 (79.6%)	0.49
Disease duration (years, mean ± SD)	15.4 ± 9.4	12.2 ± 9.7	0.34
Observation period (months), median (IQR)	35 (21–36)	36 (27–36)	0.60
RF positive, *n* (%)	9 (90.0%)	48 (88.9%)	0.92
ESR 60 (mm, mean ± SD)	69.2 ± 43.7	59.2 ± 31.2	0.40
CRP (mg/L, mean ± SD)	29.3 ± 24.7	27.7 ± 36.0	0.89
Steinbrocker stage (I + II/III + IV)	3/7	28/26	0.20
Comorbidities, *n* (%)			
Coexisting lung disease	3 (30.0%)	18 (33.3%)	0.83
Diabetes mellitus	0 (0%)	3 (5.5%)	0.44
Medications, *n* (%)			
Methotrexate	8 (80.0%)	43 (79.6%)	0.98
Other DMARDs	2 (20.0%)	19 (35.2%)	0.34
PSL (mg/day)	4.7 ± 3.2	1.3 ± 2.0	<0.001
PSL, any dose (%)	9 (90.0%)	19 (35.2%)	0.001
PSL ≧5 mg/day	6 (60.0%)	6 (11.1%)	<0.001
Biologics, *n* (%)			
TNF inhibitors	4 (40.0%)	32 (59.2%)	0.10
Tocilizumab	3 (30.0%)	3 (5.6%)	
Switch of biologics	3 (30.0%)	19 (35.2%)	

*IQR* interquartile range, *RF* rheumatoid factor, *DAS* disease activity score, *DMARDs* disease-modifying antirheumatic drugs, *PSL* prednisolone

### Low-dose glucocorticoid increases the incidence of serious infections in the biologics group of elderly RA patients

We further analyzed the risk factors of serious infections in the biologics group of elderly RA patients using multivariate logistic regression analysis (Table 4). We selected age, biologic use, glucocorticoids use, DMARD use and coexisting lung disease as independent factors for multivariate logistic regression analysis based on previous reports [3, 4, 6, 17] and our present results (Table 3). Aging over the age of 75 years was not significantly associated with serious infections in the biologics group of elderly RA patients (OR 1.1, 95% CI 0.2–5.3, *P* = 0.91). Biologics use was not significantly associated with serious infections (OR 1.1, 95% CI 0.4–3.2) in all elderly RA patients. PSL (≧5 mg/day) use was significantly associated with serious infections in the biologics group of elderly RA patients (OR 29.3, 95% CI 3.6–652.2, *P* < 0.001). Interestingly, even lower doses of PSL (1–4 mg/day) use was significantly associated with serious infections (OR 11.7, 95% CI 1.5–257.1, *P* = 0.02) in the biologics-treated patients. On the other hand, PSL (≧5 mg/day) use, but not PSL (1–4 mg/day) use, was significantly associated with serious infections in the non-biologics group of elderly RA patients (Table 4). These results suggest that even lower doses of glucocorticoid (PSL 1–4 mg/day) cause serious infections in biologics-treated patients than those do in the patients without biologics.

**Table 4:** Multiple regression analysis of risk factors for serious infections in biologics and non-biologics group of elderly RA patients

	All (*n* = 183)		Biologics (*n* = 64)		Non-biologics (*n* = 119)	
	OR (95% CI)	*P*	OR (95% CI)	*P*	OR (95% CI)	*P*
Aging ≧75 years	0.8 (0.3–2.1)	0.65	1.1 (0.2–5.3)	0.91	0.7 (0.2–2.4)	0.59
Biologics	1.1 (0.4–3.2)	0.78	–	–	–	–
PSL none	1.0 (ref.)		1.0 (ref.)		1.0 (ref.)	
1–4 mg/day	5.7 (1.5–24.2)	0.012	11.7 (1.5–257.1)	0.02	3.6 (0.4–24.5)	0.21
≧5 mg/day	21.5 (6.8–84.2)	<0.001	29.3 (3.6–652.2)	<0.001	19.2 (4.9–101.0)	<0.001
DMARDs	0.7 (0.1–6.3)	0.70	0.2 (0.01–2.7)	0.21	–	–
Coexisting lung disease	0.9 (0.3–2.5)	0.89	0.4 (0.03–2.5)	0.33	1.2 (0.3–4.3)	0.83

## Discussion

In this retrospective cohort study, we show that there is not a significant difference in the incidence of serious infections between the biologics and non-biologics groups in elderly RA patients (≧65 years; Table 2 and Fig. 1). The most interesting observation is that glucocorticoid use is the most important risk factor for serious infections in biologics-treated elderly RA patients (Table 3) and that even lower doses of glucocorticoid (PSL at <5 mg/day) increase the risk of serious infections in biologics-treated elderly RA patients, but not non-biologic DMARD-treated patients (Table 4).

We show that there is not a significant difference in the incidence of serious infections between the biologics and non-biologics groups in elderly RA patients (≧65 years). We found that, during a 3-year observation period, the incidence rate of serious infections was not significantly different between the biologics and non-biologics groups in elderly RA patients (8.0 and 6.3 events per 100 person-years of follow-up, respectively; Table 2). We also found that the time to the first serious infection did not significantly differ between the two groups by the analysis of the Kaplan–Meier curves (Fig. 1). Among serious infections, bacterial pneumonia was the most frequent infection in both the biologics and non-biologics groups (Table 2), which is in agreement with previous studies [18, 19]. In addition, there was no significant difference in the incidence of pneumocystis pneumonia between the two groups, although it has been shown that TNF inhibitors increase the incidence of opportunistic infections including pneumocystis pneumonia and tuberculosis in RA patients [20–22]. As to mycobacterium tuberculosis, there was no incidence of tuberculosis in this study. We always checked the tuberculosis screening tests before starting biologic treatment and administered isoniazid if needed.

Recent evidence that combination therapy with TNF inhibitors and MTX for RA has superior efficacy to MTX therapy also suggests the possibility of an additive risk of infections in the combination therapy for RA [23]. However, several studies have shown that the rates of overall infections and serious infections do not significantly differ between combination therapy with TNF inhibitors and MTX and MTX monotherapy for RA [6, 11, 12, 24], whereas other studies have shown that the rate of serious infections is significantly higher in the combination therapy than MTX therapy [7, 8, 10]. It has been shown that disease activity is associated with the susceptibility for hospitalized infections in RA patients [25]. Although CRP and ESR were higher in the biologics group at the start of therapy than the non-biologics group (Table 1), improvements in physical limitations with biologic agents may counteract some of the immunosuppressive effects of these drugs [26]. In addition, older ages and comorbidities are also known to be risk factors for infections in RA patients [3, 6, 27], but ages and the rate of comorbidities (coexisting lung diseases and diabetes) were not significantly different between the biologic and non-biologic groups (Table 1). All subjects in this study were old (>65 years), and it probably minimized as an effect of age for infection in this study. Therefore, our results indicate that biologic agents together with conventional DMARDs can be safely used for the treatment of elderly RA patients without increasing the risk of serious infections.

Second, our results show that even very low-dose glucocorticoid use (PSL 1–4 mg/day) significantly increases the risk of serious infections in biologics-treated elderly RA patients, but not non-biologic DMARD-treated patients. We found that PSL doses alone were significantly associated with serious infections in biologics-treated elderly RA patients (Table 3). Furthermore, we found that PSL 1–4 mg/day was associated with serious infections in biologics-treated patients, but not non-biologic DMARD-treated patients (Table 4). Our findings of the association of glucocorticoid use (PSL at >5 mg/day) with serious infections in both biologics-treated and non-biologics-treated patients (Table 4) are consistent with previous studies indicating that glucocorticoid use is the most important risk factor for serious infections in RA patients, irrespective of biologics therapy and non-biologic DMARDs therapy [3, 4, 6, 17]. Considering that 9/10 infections in the biologic cohort were on steroid, this observation deserves highlighting. The fact that our patients received very low doses of PSL (biologics 1.8 mg/day and non-biologics 1.9 mg/day) in this cohort may contribute to the present findings. Thus, our results suggest that even very low-dose glucocorticoid use (PSL 1–4 mg/day) increases the susceptibility to serious infections in biologics-treated elderly RA patients.

This study was retrospective analysis and had several limitations. This study might not have detected a difference in the incidence of serious infections between both the groups because of inadequate sample size. Actually, the calculated power of biologic agents was 6.6% in this analysis. So, to prove the hypothesis that long-term use of biologic agents does not increase the risk of serious infections in elderly RA patients as compared with non-biologic DMARDs, a larger-scale registry study or cohort study will be required in the future. On the other hand, glucocorticoids (none or use) had larger impact on serious infections (effect size *d* = 0.27) than glucocorticoids (none or use) and the calculated power was 55.0%. Glucocorticoids (PSL < 5 mg/day or PSL ≧ 5 mg/day) had larger impact on serious infections (effect size *d* = 0.40) than glucocorticoids (none or use) and the calculated power was 72.4%. Second, the patients we analyzed might have been biased samples, because we could not choose to use biologic agents for the treatment of patients with risk factors, including the known infections, respiratory dysfunction and poor performance status. Finally, the observation period was shorter in the biologics group than that in the non-biologics group (Table 2). In the biologics group, the treatment with biologic agents was stopped in the situations in which patients achieved remission or had serious infections.

In conclusion, we show that there is not a significant difference in the incidence of serious infections between the biologics and non-biologics groups in elderly RA patients (≧65 years) and that even very low-dose glucocorticoid use (PSL 1–4 mg/day) is a risk factor for serious infections in biologics-treated elderly RA patients. Although our study has some limitations because of the retrospective cohort study and a relatively small sample size, these results provide important information on the initiation and continuation of biologic therapy in elderly RA patients.
